# Supplementary material for: ExpansionHunter Denovo: a computational method for locating known and novel repeat expansions in short-read sequencing data
Source: Genome Biol. 2020 Apr 28;21:102. doi: 10.1186/s13059-020-02017-z (PMC7187524; doi:10.1186/s13059-020-02017-z)
Supplement: Supplementary file 1 — Additional file 1. Supplementary methods, supplementary results, Figure S1-S6, and captions of Tables S1-S8. [file 13059_2020_2017_MOESM1_ESM.docx]

Supplemental information

[**Supplemental Methods**](#_70aen7jecb5g) **1**

[Initial processing of WGS data](#_gwm4xdroh46e) 1

[Detection of repeat expansions](#_ql5jxun8gmpx) 2

[Baseline simulations](#_ijo458awa4qa) 3

[Comparison between STRetch and GangSTR STR databases](#_i9wc82aoqqw9) 3

[Simulation of repeat expansions](#_wuv1cjgach1i) 3

[Manual cataloging of repeats](#_ceou89wj1fi4) 4

[Validation with an orthogonal sequencing technology](#_mebenmngm2fs) 5

[Prioritization of relevant expansions](#_baol26p2mvk5) 5

[Comparison between aligners](#_uxbslzftwk3f) 6

[**Supplemental Results**](#_73hw0q1ubmjo) **7**

[Validation with an orthogonal sequencing technology](#_sbdsaaxelfel) 7

[Additional simulation results](#_71578mxdchr2) 7

[**Supplemental Figures**](#_d5a91t6wt3n5) **8**

[**Supplemental Tables**](#_qr3f4sejkuyr) **13**

# Supplemental Methods

## Initial processing of WGS data

Human and simulated WGS data was aligned to the GRCh37-lite genome

- <http://www.bcgsc.ca/downloads/genomes/9606/hg19/1000genomes/bwa_ind/genome/GRCh37-lite.fa>

with BWA-MEM v0.7.17 [[1]](https://paperpile.com/c/0if6My/5b8FC) using SAMtools v1.9 [[2]](https://paperpile.com/c/0if6My/XLio1). WGS data that was originally obtained in BAM format was converted to FASTQ using Bazam v1.0.1 [[3]](https://paperpile.com/c/0if6My/Wzmwu).

## Detection of repeat expansions

ExpansionHunter Denovo v0.8.6 was used to generate the genome-wide STR profile for each WGS sample:

ExpansionHunterDenovo profile --reads SAMPLE.bam --reference GRCh37-lite.fa --output-prefix SAMPLE

The manifest file was synthesized for each required comparison, then multisample STR profiles were generated and subsequent locus- and motif-based analyses were performed:

ExpansionHunterDenovo merge --reference GRCh37-lite.fa --manifest MANIFEST.tsv --output-prefix OUTPUT

python3 casecontrol.py locus --manifest MANIFEST.tsv --multisample-profile OUTPUT.multisample_profile.json --output-prefix casecontrol_locus.tsv

python3 casecontrol.py motif --manifest MANIFEST.tsv --multisample-profile OUTPUT.multisample_profile.json --output-prefix casecontrol_motif.tsv

python3 outlier.py locus --manifest MANIFEST.tsv --multisample-profile OUTPUT.multisample_profile.json --output-prefix outlier_locus.tsv

python3 outlier.py motif --manifest MANIFEST.tsv --multisample-profile OUTPUT.multisample_profile.json --output-prefix outlier_motif.tsv

The output of each command was sorted by p-value/z-score and the rank of each relevant locus or motif was extracted to evaluate performance.

STRetch (<https://github.com/Oshlack/STRetch>, commit 5405902) was run using the recommended pipeline for WGS analysis starting from BAM files for each sample independently (STRetch_wgs_bam_pipeline.groovy). The STRetch STR catalog was converted to GRCh37 (sed s/chr// hg19.simpleRepeat_period1-6.dedup.sorted.bed > grch37_input_regions.bed) and set as the input_regions parameter. Each sample was compared to the included controls (hg19.PCRfreeWGS_143_STRetch_controls.tsv). The rank of each relevant repeat was extracted from the sorted STRs.tsv output file.

## Baseline simulations

The 2x150bp reads were simulated using wgsim [[4]](https://paperpile.com/c/0if6My/rfGI) read simulator. The base error rate was set to 0, indel fraction to 0, and fragment length to 450 bp with a standard deviation of 50. Simulated reads were mapped to the reference genome as described above, and IRR pairs were identified from the BAM file using EHdn profile command.

## Comparison between STRetch and GangSTR STR databases

GangSTR and STRetch catalogs were downloaded on February 20th, 2019 from

- [https://s3.amazonaws.com/gangstr/hg19/genomewide/hg19_ver13_1.bed.gz](https://s3.amazonaws.com/gangstr/hg19/genomewide/hg19_ver13.bed.gz) and
- <https://figshare.com/s/1a39be9282c90c4860cd>

respectively. These catalogs were compared with the list of pathogenic loci using the Intervene tool [[5]](https://paperpile.com/c/0if6My/E0tPr) requiring at least 1 bp overlap. The comparison script, RunComparison.sh, is located here:

- https://github.com/egor-dolzhenko/ehdn-paper-analysis/tree/master/CompareSTRDatabases/

## Simulation of repeat expansions

The repeat expansions were simulated following a strategy similar to the one used by BamSurgeon [[6]](https://paperpile.com/c/0if6My/kcCeZ). Briefly, a region around a non-expanded target repeat in a control WGS sample was replaced with synthetic reads supporting an expansion in a heterozygous (one expanded allele and one reference-length allele) state (Figure S2). Specifically,

- The WGS sample HG03522 from the Polaris Kids cohort [[7]](https://paperpile.com/c/0if6My/j4XnU) was used as the control.
- A FASTA file containing the expanded repeat along with 2Kb flanking sequence upstream and downstream of the repeat was generated for read simulation.
- Reads were simulated from the FASTA file using ART v2.5.8 [[8]](https://paperpile.com/c/0if6My/7poom); read length (150bp), mean insert size (460), and insert size standard deviation (115) were chosen to match the control WGS sample.
- Processed simulated reads, same as described above, were merged with sample HG03522 using SAMtools (v1.3.1).

Further details about the simulation process can be found here:

- <https://github.com/egor-dolzhenko/ehdn-paper-analysis/tree/master/STR_Simulation>

## Manual cataloging of repeats

Pathogenic repeats were catalogued from a variety of sources including recent reviews and original discovery publications with source citations listed (Table S1). Genomic coordinates of pathogenic repeats were manually validated using IGV [[9]](https://paperpile.com/c/0if6My/UM9nd).

Twelve STRs with varying motif lengths were selected from a recently-described set of expression-linked STRs [[10]](https://paperpile.com/c/0if6My/9lOtV) (Table S2).

A set of 27 STRs with motifs of size 7-10bp were selected from the GangSTR catalog. They were chosen to overlap intronic or UTR regions of autosomal recessive genes from the OMIM catalog [[11]](https://paperpile.com/c/0if6My/0fKOW) (Table S2).

## Validation with an orthogonal sequencing technology

To validate the quality of repeat expansions detected by EHdn we obtained both short and long read sequence data for one sample and compared the calls. First, EHdn was used to detect tandem repeats in the HuRef genome [[12]](https://paperpile.com/c/0if6My/IMde3). The calls from EHdn were compared to those detected from HuRef-derived Pacific Biosciences single molecule, real-time (SMRT) long-read sequencing data. Specifically, EHdn was run on Illumina HiSeq X reads from DNA extracted from HuRef blood (NCBI sequence read archive accession SRR9046649). The sequence reads were aligned to GRCh38/hg38. Insertions and deletions were detected from the SMRT data by performing *de novo* assembly using Canu [[13]](https://paperpile.com/c/0if6My/I53Hy) followed by variant detection with AsmVar (<https://github.com/bioinformatics-centre/AsmVar>). Because EHdn detects repeats greater than the read length (in this case, 150 bp), an EHdn call was considered validated if the size of the largest overlapping Tandem Repeats Finder (TRF) [[14]](https://paperpile.com/c/0if6My/dMSfZ) repeat plus the size of any AsmVar insertion (positive) or deletion (negative) was >=150 bp. For instance, if an EHdn call overlapped a TRF region of size 80 bp and AsmVar detected a 100 bp insertion overlapping the TRF region, then the total tandem repeat size would be 180 bp, validating the EHdn call. Conversely, the EHdn region would not be considered validated if the overlapping TRF region was 200 bp and there was a 60 bp AsmVar deletion, giving a total size of 140 bp. If an EHdn region did not overlap a TRF region, but it overlapped an AsmVar insertion >=150 bp, then it was considered validated. TRF regions were padded by 100 bp on either side for the purposes of determining overlap with AsmVar variants.

## Prioritization of relevant expansions

We obtained NCBI gene IDs for 16,227 brain-expressed genes from the Human Protein Atlas ([http://www.proteinatlas.org](https://www.proteinatlas.org/search/NOT+tissue_category_rna%3Abrain%3Bnot+detected)) [[15]](https://paperpile.com/c/0if6My/42MEY) and then mapped them to GRCh37/hg19 exon boundaries via HGNC IDs [[16]](https://paperpile.com/c/0if6My/piCSw).

For each repeat (DMPK, FXN, FMR1, and HTT) we generated 1,000 datasets containing five samples with expansions and 150 controls by repeatedly selecting five random samples from the set of all available samples with that expansion. After running the outlier analysis on each of the resulting datasets, we ranked the regions by the number of samples reported in the "high_case_counts" column.

## Comparison between aligners

We evaluated the performance of ExpansionHunter Denovo when applied to reads mapped by several commonly used aligners. This comparison was performed using sample HG00096 from the Polaris Diversity cohort using the GRCh37-lite genome and comparing the following aligners: Isaac (v03.16.02.19) [[17]](https://paperpile.com/c/0if6My/dCjPE), BWA-MEM (v0.7.17) [[1]](https://paperpile.com/c/0if6My/5b8FC), bowtie2 (v2.3.5.1) [[18]](https://paperpile.com/c/0if6My/Ou2Pn), hisat2 (v2.1.0) [[19]](https://paperpile.com/c/0if6My/ljx1C) and minimap2 (v2.17) [[20]](https://paperpile.com/c/0if6My/059us). All aligners were run using default parameters.

EHdn (v0.8.6) “profile” command was run on the aligned bam files using default parameters except for the bowtie2 alignments, which have a maximum MAPQ value of 44, thus the “min-anchor-mapq” and “max-irr-mapq” parameters were adjusted from their default values of 40 and 50, respectively, and both set equal to 30.

The correlation between STR profiles generated by EHdn for reads mapped by each aligner was computed for both anchored-IRRs and paired-IRRs by merging the STR profiles for each pair of aligners and computing Pearson’s correlation coefficient between the two samples across all merged regions and motifs.

# Supplemental Results

## Validation with an orthogonal sequencing technology

EHdn detected 374 tandem repeats in the HuRef genome having at least five anchored in-repeat reads. A total of 292 of them were validated using a combination of Tandem Repeats Finder reference repeats and insertions and deletions detected from PacBio data, giving a validation rate of 78%. As the minimum number of anchored in-repeat reads was increased, the validation rate improved modestly, but the total number of calls dropped substantially (Figure S4).

## Additional simulation results

Since we anticipate that EHdn will be used for discovery of novel pathogenic REs, we additionally simulated expansions of STRs with an associated effect on gene expression [[10]](https://paperpile.com/c/0if6My/9lOtV). From the catalog of fine-mapped eSTRs, we selected 12 loci with varying motifs linked with known disease genes. Expansions with 200 copies were simulated at these loci and EHdn prioritized all 12 REs in the top five. STRetch also performed well at these loci, although missed three because they were not present in its catalog.

To highlight that EHdn is not limited to short STR motifs, we tested its capacity to detect an expansion of a known pathogenic repeat with 12 bp motif in the promoter region of the *CSTB* gene. Using EHdn we detected this simulated expansion at the pathogenic lower bound (40 copies) (Table S6). We further demonstrate the ability of our method to detect REs with longer motifs at other similar loci. We simulated expansions of 27 repeats with 7-10 bp motifs within genes implicated in autosomal recessive genetic diseases (Supplemental Methods). All 27 loci were ranked in the top five in both the locus and motif analyses (Table S6).

# Supplemental Figures


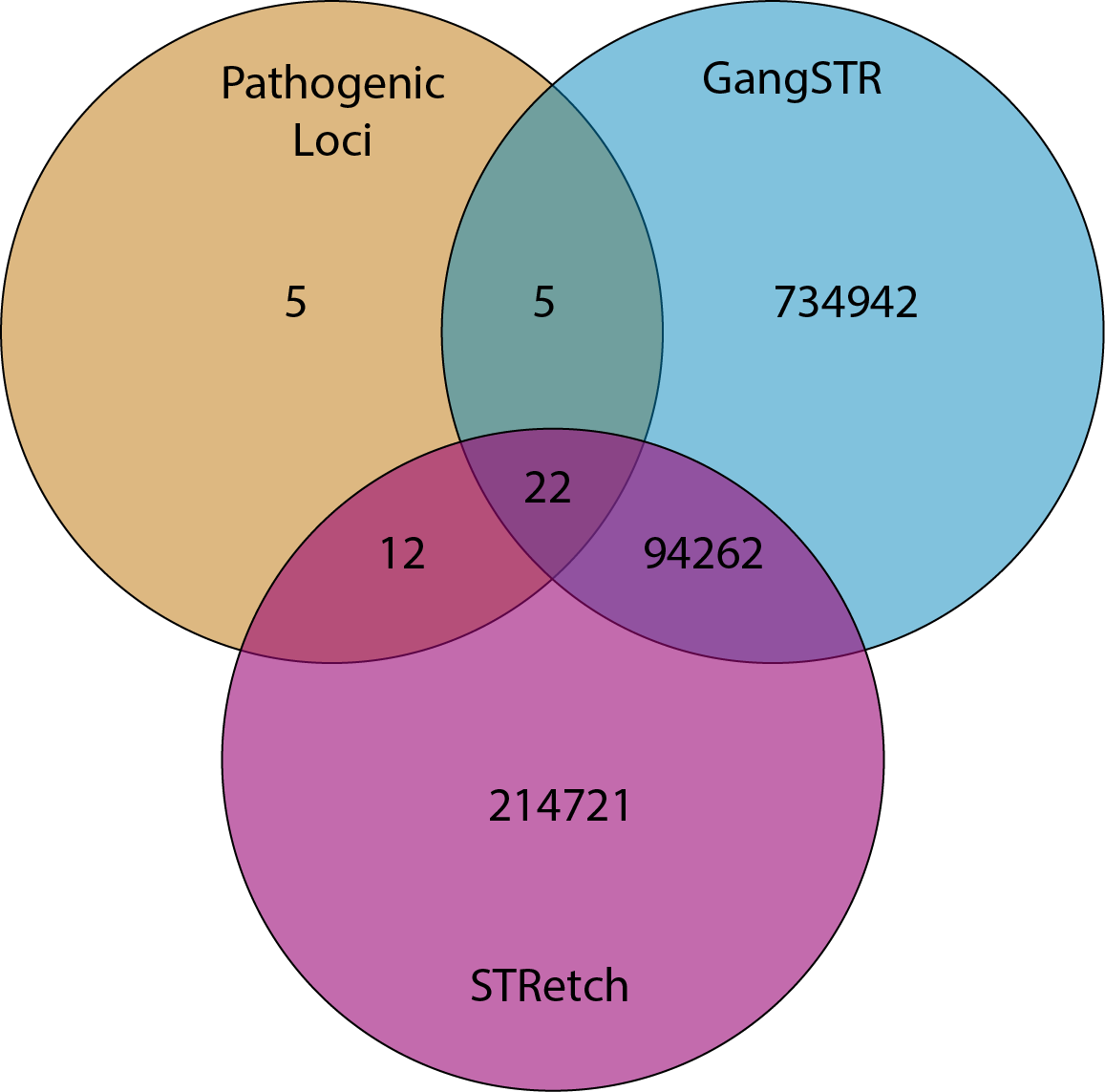


Figure S1: Summary of concordance between STR catalogs: STRetch catalog (based on the UCSC simple repeats track), the GangSTR catalog, and a curated list of pathogenic or potentially pathogenic repeats reported in the literature.


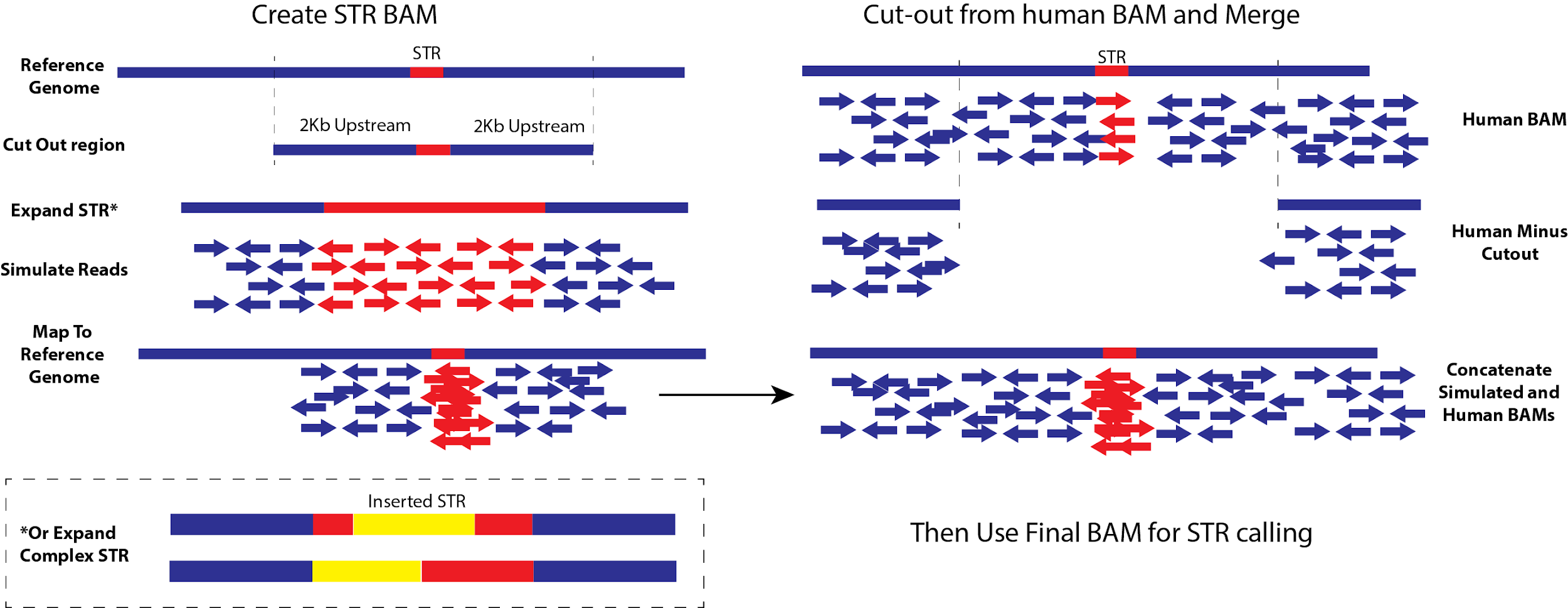


Figure S2: Overview of simulating samples with repeat expansions. STR simulation by replacing reads around a non-expanded repeat in a control WGS sample with synthetic reads supporting an expansion.


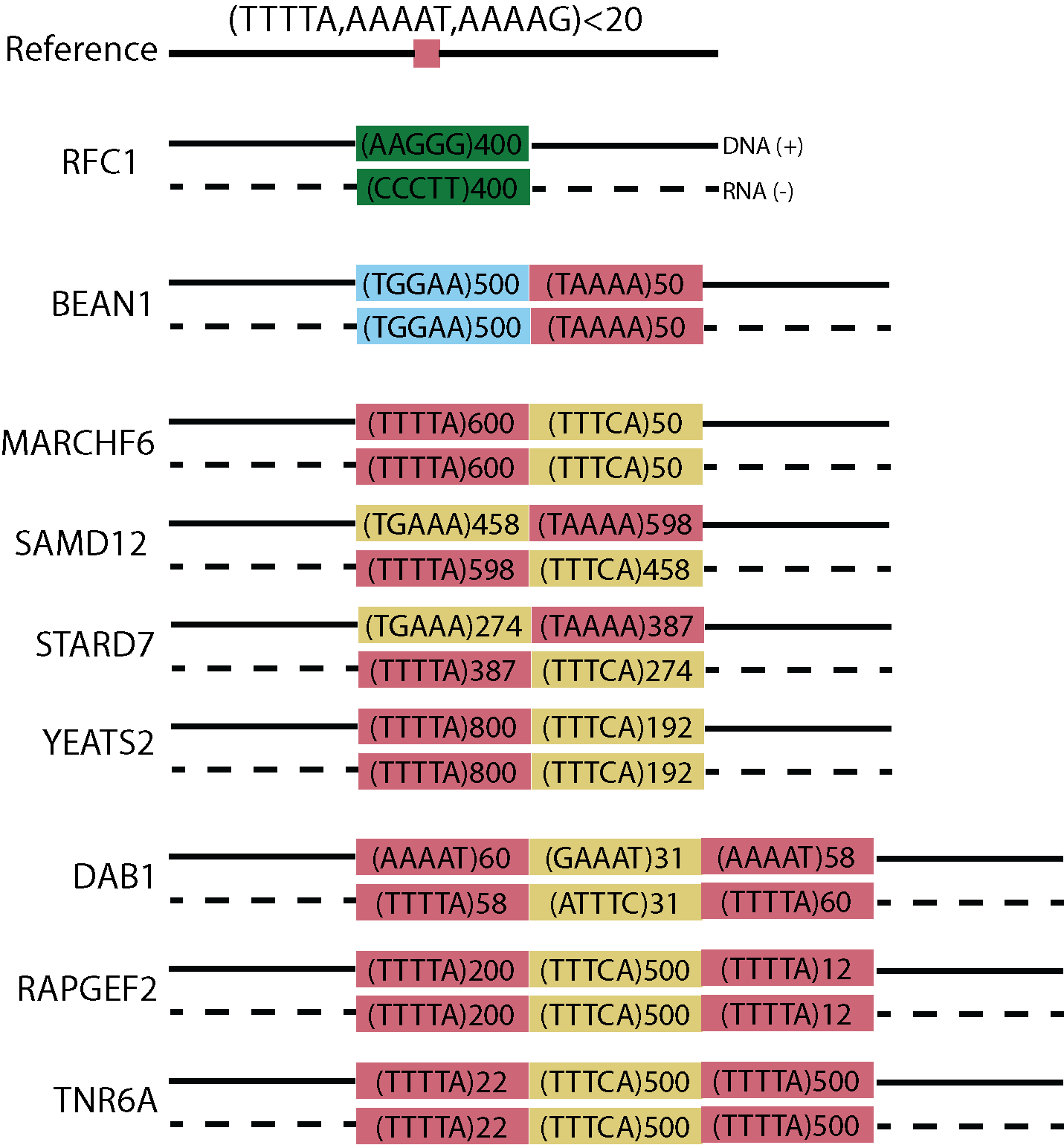


Figure S3: Structure of nine complex pathogenic repeats.

**
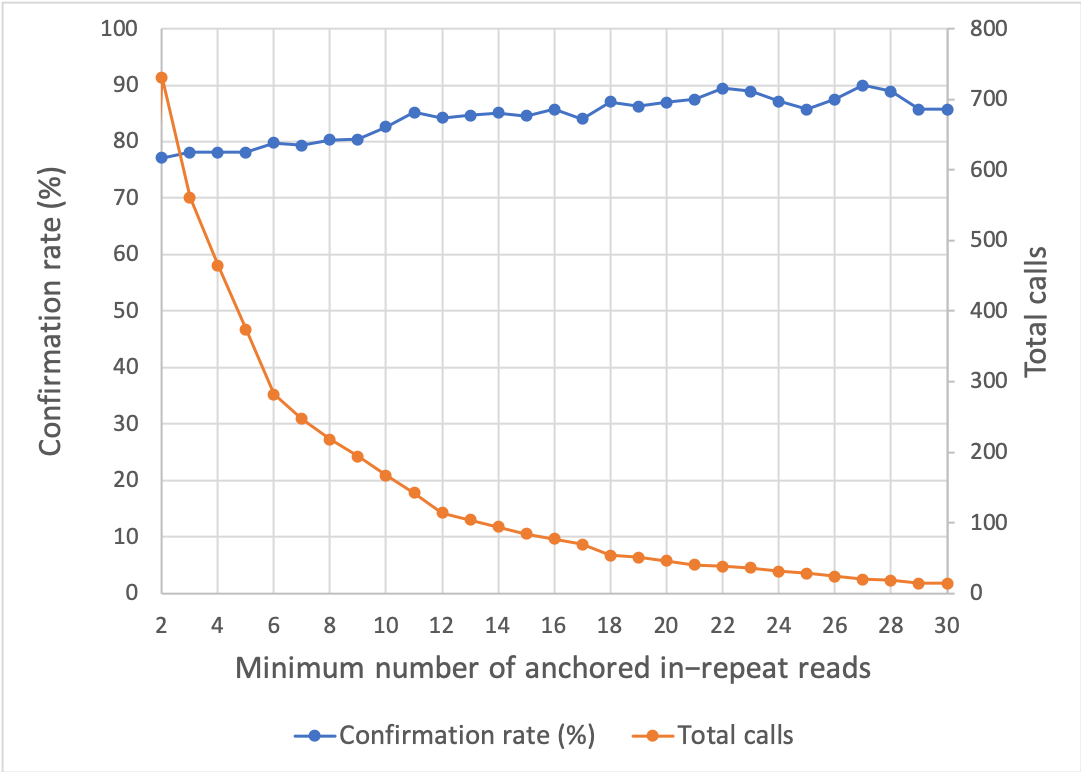
**

Figure S4: Effect of the minimum number of anchored in-repeat reads on the confirmation rate and total number of calls from EHdn.


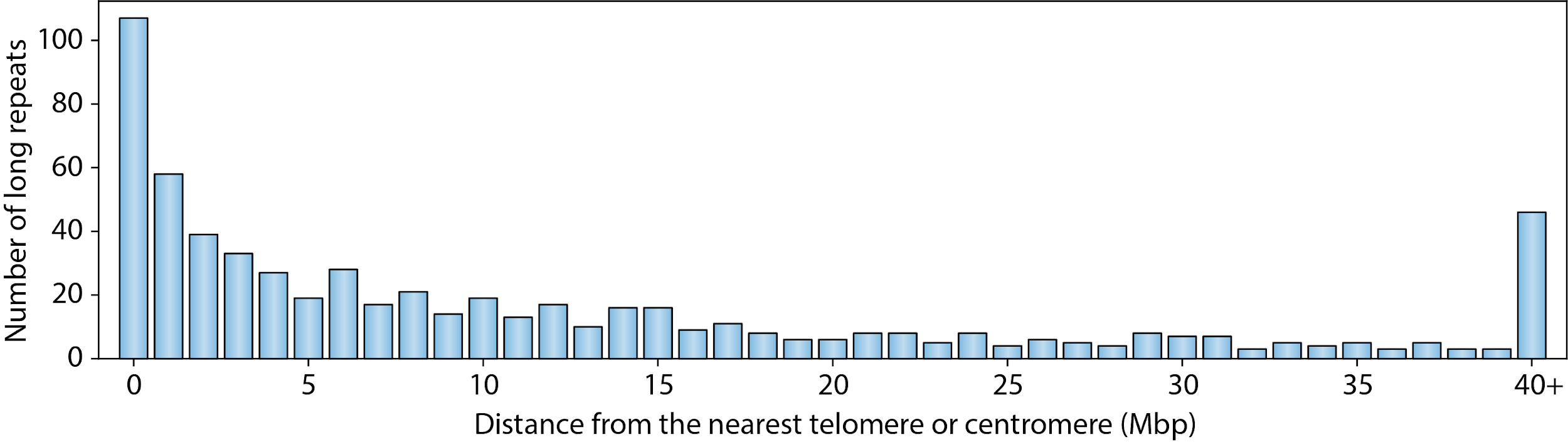

Figure S5: Proximity (in megabase pairs) of long (150-200bp and longer) repeats identified in the control population to the closest telomere/centromere.


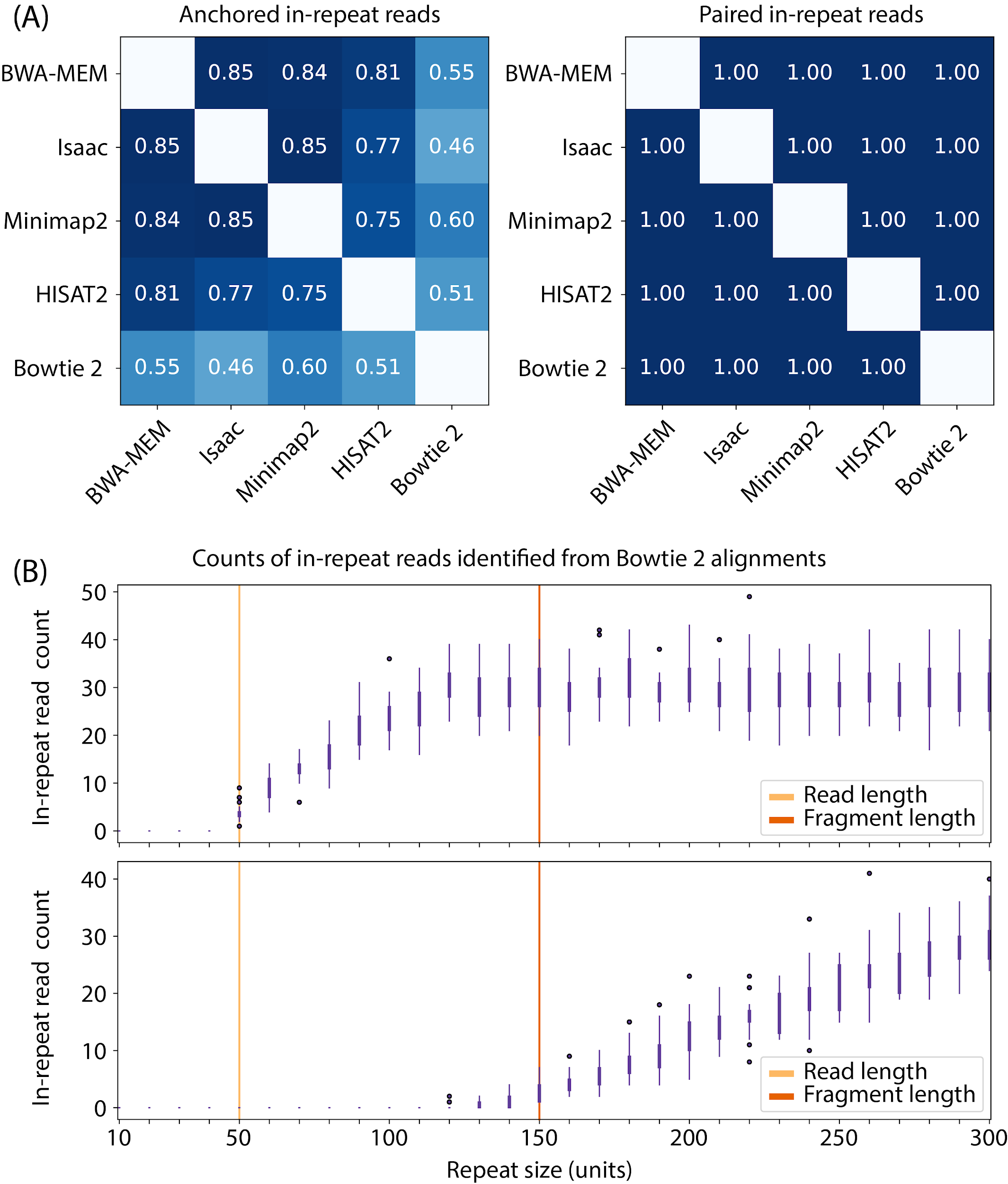


Figure S6: (A) Correlation between STR profiles for sample HG00096 generated from alignments obtained with a variety of aligners. (B) The counts of in-repeat reads recovered from simulated reads for a variety of repeat sizes and aligned with Bowtie 2.

# Supplemental Tables

Table S1 (Additional file 2)

Definitions of small, large, degenerate, and complex repeat expansions. Gene, publication source, GRCh37 and GRCh38 reference coordinates in BED format (0-based half-open), repeat motif, pathogenic lower bound, motif in frame of gene, and presence in STRetch/GangSTR databases is listed for each repeat locus. For degenerate repeats, ambiguous nucleotides are represented with ‘N’ and the locus is defined with respect to the amino-acid expansion (e.g. poly-Alanine).

Table S2 (Additional file 3)

Repeats with long motifs (motif length 7-10bp) and repeat loci linked to gene expression which were used for simulation. Associated gene, genic region (e.g. intronic, upstream, UTR5), GRCh37 coordinates, repeat motif, and simulated size in motif counts are provided for each locus.

Table S3 (Additional file 4)

Results for simulations of 13 small pathogenic repeat expansion loci for STRetch, EHdn Locus, and EHdn Motif outlier prioritization analyses. Expansions are shown for a range of sizes larger than the pathogenic lower bound and exceeding 150bp. Each row describes a single simulation of a repeat expansion, with the gene, pathogenic lower bound, motif, motif length, simulated size in copies, simulated size in base pairs, the STR tool which was used, the rank, the STR rank (only comparing motifs length 2-6bp), and the Z-score or p-value are listed. Missing values are represented by -1.

Table S4 (Additional file 5)

Results for simulations of 22 large pathogenic repeat expansions for STRetch, EHdn Locus, and EHdn Motif outlier prioritization analyses. Expansions are shown for a range of sizes larger than the pathogenic lower bound and exceeding 150bp. Columns shown are described in Table S3. Missing values are represented by -1.

Table S5 (Additional file 6)

Results for simulations of 27 large-motif repeat expansions for EHdn Locus and EHdn Motif outlier prioritization analyses. Each repeat consists of 100 motif copies (motif length 7-10bp). Both EHdn motif and EHdn locus prioritization results are shown. Columns shown are described in Table S3. Missing values are represented by -1.

Table S6 (Additional file 7)

Results for simulations of small-motif expression-linked repeat expansions. Each repeat consists of 200 motif copies (motif length 2-6 bp). STRetch, EHdn motif, and EHdn locus prioritization results are shown. Columns shown are described in Table S3. Missing values are represented by -1.

Table S7 (Additional file 8)

Results for simulations of nine complex pathogenic repeat expansions with EHdn Locus and EHdn Motif outlier prioritization analyses. Each locus is delineated by gene name, such that a single simulation is performed for each gene. Ranks for each motif present at a given locus are shown as separate rows annotated by sub-motif. Ranks are additionally listed for both outlier analysis performed with the parameter defining the maximum mapping quality threshold for IRRs (--max-irr-mapq) set to 40 (default) and 60. Missing values are represented by ‘N/A’.

Table S8 (Additional file 9)

Overview of existing methods for detecting repeat expansions with short-read data.

[1. Li H. Aligning sequence reads, clone sequences and assembly contigs with BWA-MEM. arXiv [q-bio.GN]. 2013. Available from:](http://paperpile.com/b/0if6My/5b8FC) <http://arxiv.org/abs/1303.3997>

[2. Li H, Handsaker B, Wysoker A, Fennell T, Ruan J, Homer N, et al. The Sequence Alignment/Map format and SAMtools. Bioinformatics. 2009;25:2078–9.](http://paperpile.com/b/0if6My/XLio1)

[3. Sadedin SP, Oshlack A. Bazam: a rapid method for read extraction and realignment of high-throughput sequencing data. Genome Biol. 2019;20:78.](http://paperpile.com/b/0if6My/Wzmwu)

[4. Li H. lh3/wgsim. GitHub. Available from:](http://paperpile.com/b/0if6My/rfGI) <https://github.com/lh3/wgsim>

[5. Khan A, Mathelier A. Intervene: a tool for intersection and visualization of multiple gene or genomic region sets. BMC Bioinformatics. 2017;18:287.](http://paperpile.com/b/0if6My/E0tPr)

[6. Ewing AD, Houlahan KE, Hu Y, Ellrott K, Caloian C, Yamaguchi TN, et al. Combining tumor genome simulation with crowdsourcing to benchmark somatic single-nucleotide-variant detection. Nat Methods. 2015;12:623–30.](http://paperpile.com/b/0if6My/kcCeZ)

[7. Illumina, Inc. Illumina/Polaris. GitHub. Available from:](http://paperpile.com/b/0if6My/j4XnU) <https://github.com/Illumina/Polaris>

[8. Huang W, Li L, Myers JR, Marth GT. ART: a next-generation sequencing read simulator. Bioinformatics. 2012;28:593–4.](http://paperpile.com/b/0if6My/7poom)

[9. Robinson JT, Thorvaldsdóttir H, Winckler W, Guttman M, Lander ES, Getz G, et al. Integrative genomics viewer. Nat Biotechnol. 2011;29:24–6.](http://paperpile.com/b/0if6My/UM9nd)

[10. Fotsing SF, Margoliash J, Wang C, Saini S, Yanicky R, Shleizer-Burko S, et al. The impact of short tandem repeat variation on gene expression. Nat Genet. 2019;51:1652–9.](http://paperpile.com/b/0if6My/9lOtV)

[11. OMIM - Online Mendelian Inheritance in Man. Available from:](http://paperpile.com/b/0if6My/0fKOW) <https://omim.org/>

[12. Levy S, Sutton G, Ng PC, Feuk L, Halpern AL, Walenz BP, et al. The diploid genome sequence of an individual human. PLoS Biol. 2007;5:e254.](http://paperpile.com/b/0if6My/IMde3)

[13. Koren S, Walenz BP, Berlin K, Miller JR, Bergman NH, Phillippy AM. Canu: scalable and accurate long-read assembly via adaptive k-mer weighting and repeat separation. Genome Res. 2017;27:722–36.](http://paperpile.com/b/0if6My/I53Hy)

[14. Benson G. Tandem repeats finder: a program to analyze DNA sequences. Nucleic Acids Res. 1999;27:573–80.](http://paperpile.com/b/0if6My/dMSfZ)

[15. Uhlén M, Fagerberg L, Hallström BM, Lindskog C, Oksvold P, Mardinoglu A, et al. Proteomics. Tissue-based map of the human proteome. Science. 2015;347:1260419.](http://paperpile.com/b/0if6My/42MEY)

[16. Yates B, Braschi B, Gray KA, Seal RL, Tweedie S, Bruford EA. Genenames.org: the HGNC and VGNC resources in 2017. Nucleic Acids Res. 2017;45:D619–25.](http://paperpile.com/b/0if6My/piCSw)

[17. Raczy C, Petrovski R, Saunders CT, Chorny I, Kruglyak S, Margulies EH, et al. Isaac: ultra-fast whole-genome secondary analysis on Illumina sequencing platforms. Bioinformatics. 2013;29:2041–3.](http://paperpile.com/b/0if6My/dCjPE)

[18. Langmead B, Salzberg SL. Fast gapped-read alignment with Bowtie 2. Nat Methods. 2012;9:357–9.](http://paperpile.com/b/0if6My/Ou2Pn)

[19. Kim D, Langmead B, Salzberg SL. HISAT: a fast spliced aligner with low memory requirements. Nat Methods. 2015;12:357–60.](http://paperpile.com/b/0if6My/ljx1C)

[20. Li H. Minimap2: pairwise alignment for nucleotide sequences. Bioinformatics. 2018;34:3094–100.](http://paperpile.com/b/0if6My/059us)
