# Supplementary material for: ExpansionHunter Denovo: a computational method for locating known and novel repeat expansions in short-read sequencing data
Source: Genome Biol. 2020 Apr 28;21:102. doi: 10.1186/s13059-020-02017-z (PMC7187524; doi:10.1186/s13059-020-02017-z)
Supplement: Supplementary file 10 — Additional file 10. Review history. [file 13059_2020_2017_MOESM10_ESM.docx]

**First round**

**Reviewer 1**

The paper describes a tool for detecting repeat expansions from short read data. As these data are now ubiquitous this tool is useful for a number of researchers. There are however a number of other tools able to solve the same problem and it is not clear what the benefit of this tool over those tools is.

Comments:

1) The manuscript lists as the tools benefits that it does not require a catalog of known variants. They state that most of the time the pathogenic expansion will be in the top ten list of reported expansions. This will be cumbersome for the user and some more guidance might be useful. It would be very beneficial to add some information to the ranking of repeat expansion. Is annotation considered, i.e. coding, intronic, up/downstream etc.?

2) Does EHdn predict the number of repeats at the locations it reports, like in Figure 1?

3) Smart to lower the penalty of mismsatch low-quality bases in the weighted purity score computation.

4) What happens to read pairs when both are IRRs but with a different motif?

5) In the general population analysis, each person is reported to have on average 658 long repeats. What is the limit for a long repeat and what fraction of these are non-telo/centromeric?

6) Repeat expansions are generally easier to find from long read data, the authors should make this clear to the reader.

7) Is the tool called EHdn (as the discussion suggests) or ExpansionHunter Denovo (as the title suggests)

I did not seriously attempt installation of the software or running it. I read the installation instructions and they seemed reasonable. The link to releases (https://github.com/egor-dolzhenko/ExpansionHunterDenovo/releases) pointed to here (https://github.com/Illumina/ExpansionHunterDenovo/blob/master/documentation/02_Installation.md) is not correct. Replacing "egor-dolzhenko" with "Illumina" gives the correct path.

**Reviewer 2**

In this paper, Dolzhenko et al describe a third iteration of ExpansionHunter, to detect repeat expansions (REs) following two previous versions publications in Genome Research (2017) and Bioinformatics (2019). REs are increasingly being implicated in a variety of human diseases however they are challenging to detect using short read technologies. The first version was designed to call REs longer than read length, the second version was reimplemented to use sequence graphs to capture more complex STR loci using a pre-defined catalog of genomic locations, while this version's major advancement is the removal of the need for this pre-defined catalog.

For this review, I have both read the manuscript and downloaded and run the software from github. Overall, the software installs and runs easily and manuscript reads wells and appears to offer additional functionality compared to existing tools however I have several major concerns.

Major:

1)	How sensitivite is ExpansionHunter de novo (EHdn) to the choice of aligner and aligner parameters? I suspect aligner choice may impact the performance of EHdn due to aligner differences in their handling of both mapping in low-complexity regions and truncated alignments (hard/soft clipped reads).

2)	While this iteration of ExpansionHunter (EH) addresses one of the major limitations of previous EH versions and existing RE software (namely the requirement for pre-defined expansion catalogues), I am not fully convinced this iteration warrants an additional publication. While a new github repository was created for this version, a large portion of the code simply notes "Transfer code from an internal repository" and the amount of novel code written is unclear. Can the authors more clearly demonstrate how this differs from the previous ExpansionHunter iterations and what new code was written for this version?

3)	Each sample is analysed individually and in-repeat reads (IRRs) merged in the subsequent step if within 500bp of each other. Did the authors try running EHdn across all samples simultaneously (similar to GATK group variant calling)? This may improve results given the likelihood of low-quality mapping scores in these low complexity expansion regions.

4)	Description and usage of the case control vs outlier use cases seem overly complicated to me. Is it not simpler to assume with multiple "case" samples you look for shared REs while a single "case" sample runs the outlier analysis? An option to override this default behaviour could be offered for cases such as C9orf72.

5)	The final output consists solely of two summary tsv files. Given the potential for false positives, users ideally should be able to investigate all the reads that went into making this call. Many other variant detection tools (particularly SV tools) create new BAM files for each event allowing users to examine the evidence themselves however I appreciate in this instance many reads will be unaligned. While a constructed BAM file would be preferable, at a minimum a list of supporting reads used for each RE call should be provided.

6)	Is there any handling of base calling errors within repeats? Given that the error rate will likely be higher than normal in these low complexity regions and the repeats potentially very long, is this handled and if so how?

7)	Several other RE software packages are mentioned throughout the text for comparison noting limitations, differences, etc. It would help clarify where EHdn fits in with existing software (and previous EH iterations) by providing a comprehensive table highlighted the novelty of EHdn.

8)	EHdn is designed to detect expansions larger than the read size however presumably users are interested in detecting all expansions independent of size. Are there plans to handle this use case or else other tools to recommend such as previous EH versions?

9)	Most NGS sequencing technologies perform sub-optimally in high/low GC content regions which are often present in expansions. In your analysis what REs were validated using Sanger sequencing? Were any of the control population REs validated?

10)	What happens if the same repeat expansion pattern is present at two distinct genomic locations? Would this be reported as one or two events? How will paired IRRs be handled in this case? Will these be counted twice?

Minor:

1)	The output files columns are not described in the documentation. While mostly intuitive, these should be described. Also, is it possible to output a more widely acceptable format like vcf as well?

2)	Fig 3: In addition to the four known expansions, there appear to be other significant regions. Were these investigated and if so what was found?

3)	For users working with increasingly large number of WGS samples it would be useful to have the option to run EHdn on targeted regions. Is it possible to add this feature?

4)	Are controls samples required to run the EHdn?

5)	Typo P16 L4 "of motif not present the reference genome"

**Response to reviewers**

We would like to thank both reviewers for the insightful comments. We have revised the manuscript according to your suggestions. Here is a summary of the significant changes:

- Shown that ranking of the relevant repeat expansions can be significantly improved by restricting the analysis to the relevant genomic regions or by using a larger set of cases

- Implemented a script for annotating regions reported by EHdn

- Shown that EHdn can identify repeat expansions from BAMs produced by a number of short-read aligners

- Made numerous improvements to the online documentation

- Enabled EHdn to produce additional output files with repeat size estimates and information about all informative reads

- Confirmed that long repeats detected by EHdn are supported by PacBio long-read data

- Stratified repeats in the control cohort by their proximity to telomere/centromere regions

Unfortunately, while performing additional analysis we noticed that our original analysis included a couple of truncated BAMs missing data for some chromosomes. We apologize for this error and have repeated the analyses. The results are almost entirely unchanged, with a slight improvement in some rankings.

> Reviewer #1: The paper describes a tool for detecting repeat expansions from short read data. As these data are now ubiquitous this tool is useful for a number of researchers. There are however a number of other tools able to solve the same problem and it is not clear what the benefit of this tool over those tools is.

Thank you very much for the constructive feedback. Your comments helped us to significantly improve the manuscript, the program, and the documentation. Below please find detailed responses to your queries.

Although there are many great tools for STR analysis, they are designed for targeted analysis of specified STRs catalogs. We have developed ExpansionHunter Denovo in response to a common request from the users of the original ExpansionHunter to provide a way for performing efficient genome-wide scans to identify novel expansions.

A recently published paper [1] highlights some practical advantages of ExpansionHunter Denovo over other existing tools. Namely that EHdn is the only currently available computational method capable of discovering the emerging class of non-reference pathogenic expansions. We have clarified the wording in the manuscript to highlight how EHdn fills a need not provided by the other methods.

[1] Rafehi et al., Bioinformatics-Based Identification of Expanded Repeats: A Nonreference Intronic Pentamer Expansion in RFC1 Causes CANVAS, AJHG, 2019 Jul 3;105(1):151-165, <https://www.ncbi.nlm.nih.gov/pubmed/31230722>

> Comments:

>

> 1) The manuscript lists as the tools benefits that it does not require a catalog of known variants. They state that most of the time the pathogenic expansion will be in the top ten list of reported expansions. This will be cumbersome for the user and some more guidance might be useful. It would be very beneficial to add some information to the ranking of repeat expansion. Is annotation considered, i.e. coding, intronic, up/downstream etc.?

Thank you for suggesting this. We have implemented a script that annotates regions identified by EHdn with annotations from the ANNOVAR database. It is documented here:

https://github.com/Illumina/ExpansionHunterDenovo/blob/master/documentation/08_An notation.md

We now also highlight a number of practical prioritization strategies in the manuscript and online documentation:

- We show that after restricting the analysis to expansions located near exons of brainexpressed genes, most pathogenic expansions get ranked near the top (Figure 4C).

This choice of genes is motivated by the fact that nearly all known pathogenic repeats cause neurological disorders and occur in genes expressed in the brain.

- Our previous analyses described the worst-case situation of comparing a single case sample to a set of controls. We now show that when multiple cases harbor the same repeat expansion (we considered the case of N=5), nearly all pathogenic expansions get the top rank (Figure 4D).

- The vast majority of z-scores corresponding to rare expansions in the control population are 10 and below while large pathogenic expansions yield much higher zscores: https://github.com/Illumina/ExpansionHunterDenovo/blob/master/documentation/09_Re

peat_prioritization_strategies.md

Taken together, these prioritization strategies show that the ranking can be improved by either obtaining a larger set of cases or by restricting the analysis to the relevant genomic regions. This will be of benefit to the user.

> 2) Does EHdn predict the number of repeats at the locations it reports, like in Figure 1?

This is a great idea. We have extended the “profile” command to output supplementary files containing an estimate of the repeat size and depth-normalized counts of in-repeat reads. Thank you for suggesting this change.

The format of these supplementary files is described here:

https://github.com/Illumina/ExpansionHunterDenovo/blob/master/documentation/05_Co

mputing_profiles.md#supplementary-files-generated-by-the-profile-command

> 3) Smart to lower the penalty of mismatch low-quality bases in the weighted purity score computation.

Thank you! A lot of progress has been made on improving data quality in STR regions so hopefully measures like the weighted purity score would one day become obsolete.

> 4) What happens to read pairs when both are IRRs but with a different motif?

This is a very interesting question. Currently, the program does not include information about such IRR pairs in the STR profile. However, it is clear that such read pairs are of interest. To provide users with a way to access this information, EHdn now reports such IRR pairs in the log file containing information about all informative reads. See documentation for the description of this file (output_prefix.reads.tsv):

https://github.com/Illumina/ExpansionHunterDenovo/blob/master/documentation/05_Co

mputing_profiles.md#supplementary-files-generated-by-the-profile-command

We are continuing to improve EHdn and future versions of the program will include better assessment and classification of such IRRs.

> 5) In the general population analysis, each person is reported to have on average 658 long repeats. What is the limit for a long repeat and what fraction of these are nontelo/ centromeric?

We defined long repeats as repeats supported by at least five in-repeat reads which correspond to repeats starting at about 150-200 base pairs and longer (this information is now included in the manuscript). To estimate the number of repeats located outside of the telomere and centromere regions, we stratified the repeats by their distance to the closest telomere/centromere (Figure S5). We found that, on average, about a quarter of repeats are located closer than 2 Mbp to the nearest telomere/centromere and about a third were located further than 15 Mbp. We have updated the section “The landscape of long repeats within a control population” to include this additional information about where these repeats are found.

> 6) Repeat expansions are generally easier to find from long read data, the authors should make this clear to the reader.

We agree and have added text to the Discussion section to emphasize that repeat expansions are easier to identify in long read data. We have also emphasized that large cohorts of samples are already sequenced with high-throughput (e.g. short read) data and that is the driver for tools such as EHdn. It is anticipated that due to the existing cost differential between short and long-read sequencing, large cohorts will continue to be generated with short-read sequencing for some time.

> 7) Is the tool called EHdn (as the discussion suggests) or ExpansionHunter Denovo (as the title suggests)

The official name of the tool is ExpansionHunter Denovo. During the course of development we settled on the EHdn abbreviation. We have revised the manuscript to make it clear that EHdn is an abbreviation (In particular, we added EHdn to the list of abbreviations at the end of the paper.)

> I did not seriously attempt installation of the software or running it. I read the installation instructions and they seemed reasonable. The link to releases <urldefense link> pointed to here <urldefense link> is not correct. Replacing "egor-dolzhenko" with "Illumina" gives the correct path.

Thank you for noticing this. We have updated the link.

> Reviewer #2: In this paper, Dolzhenko et al describe a third iteration of ExpansionHunter, to detect repeat expansions (REs) following two previous versions publications in Genome Research (2017) and Bioinformatics (2019). REs are increasingly being implicated in a variety of human diseases however they are challenging to detect using short read technologies. The first version was designed to call REs longer than read length, the second version was reimplemented to use sequence graphs to capture more complex STR loci using a pre-defined catalog of genomic locations, while this version's major advancement is the removal of the need for this pre-defined catalog.

>

> For this review, I have both read the manuscript and downloaded and run the software from github. Overall, the software installs and runs easily and manuscript reads wells and appears to offer additional functionality compared to existing tools however I have several major concerns.

We appreciate you trying out the ExpansionHunter Denovo software and we are grateful for the constructive feedback and suggestions which have helped us improve the manuscript and program. We have already received positive feedback from one of our users about your suggestion to provide a file summarizing the informative reads. We are pleased to hear that you are aware of our previous work. We plan to continue maintaining both the original ExpansionHunter and ExpansionHunter Denovo as two independent tools serving related yet distinct purposes. ExpansionHunter was designed to target known repeat loci, using a pre-defined catalog of genomic locations. The new method, ExpansionHunter Denovo, is intended to complement the existing method, as it is designed to search genome-wide for expansions without the need for a pre-defined catalog. We hope that ExpansionHunter Denovo will enable researchers discover novel pathogenic repeat expansions in large sequencing datasets, which can then be followed up using the existing targeted methods once their genome location has been identified.

> Major:

>

> 1) How sensitive is ExpansionHunter de novo (EHdn) to the choice of aligner and aligner parameters? I suspect aligner choice may impact the performance of EHdn due to aligner differences in their handling of both mapping in low-complexity regions and truncated alignments (hard/soft clipped reads).

Thank you for raising this important question. To investigate genome-wide consistency between aligners, we compared STR profiles generated by EHdn from reads aligned with BWA MEM, Isaac, Bowtie 2, HISAT2, and Minimap2. The results were highly correlated (Figure S6A) for all aligners except Bowtie 2. EHdn recovered fewer anchored in-repeat reads from the Bowtie 2 BAM file because Bowtie 2 by default performs end-to-end alignment that involves all bases in a read. Thus Bowtie 2 is less efficient at aligning anchors that have a significant overlap with the expanded repeat.

Despite the lower efficiency, EHdn can detect expanded repeats in Bowtie 2 BAMs quite well (Figure S6B). As for any other comparitive analysis, these results underscore that it is very important to use the same aligner for the entire dataset and we now highlight this fact in the documentation:

https://github.com/Illumina/ExpansionHunterDenovo/blob/master/documentation/01_Be

fore_you_begin.md

> 2) While this iteration of ExpansionHunter (EH) addresses one of the major limitations of previous EH versions and existing RE software (namely the requirement for pre-defined expansion catalogues), I am not fully convinced this iteration warrants an additional publication. While a new github repository was created for this version, a large portion of the code simply notes "Transfer code from an internal repository" and the amount of novel code written is unclear. Can the authors more clearly demonstrate how this differs from the previous ExpansionHunter iterations and what new code was written for this version?

Apologies for not making the development process more transparent. The work on EHdn began inside a repository on Illumina’s own self-hosted GitHub instance. (This is what we refer to as an “internal repository”.) Once we received permission to make the software open-source, we transferred the code to the public GitHub. To provide some insight into the development history of EHdn we included a file with the commit history of the internal repository with the initial commit dating back to Sep. 2016. We decided to develop the two programs independently because of their different purpose: ExpansionHunter is made for accurate analysis of small numbers of pathogenic repeats while ExpansionHunter Denovo is made to rapidly detect signatures of long expansions in large sequencing datasets. Since ExpansionHunter Denovo is not meant to replace ExpansionHunter we plan to continue maintaining and improving both programs.

> 3) Each sample is analysed individually and in-repeat reads (IRRs) merged in the subsequent step if within 500bp of each other. Did the authors try running EHdn across all samples simultaneously (similar to GATK group variant calling)? This may improve results given the likelihood of low-quality mapping scores in these low complexity expansion regions.

This is a very interesting idea. Although the current version of EHdn can generate STR profiles only for one sample at a time, we believe that scanning alignments in multiple samples simultaneously would improve the accuracy of the program. We will definitely explore this idea in the future. Seeing that EHdn is now being used to analyze datasets containing tens of thousands of samples (which would be costly to process simultaneously) we are exploring the possibility of implementing a reanalysis tool that would reanalyze regions flagged by EHdn by scanning the alignments in a subset of samples from the original dataset.

> 4) Description and usage of the case control vs outlier use cases seem overly complicated to me. Is it not simpler to assume with multiple "case" samples you look for shared REs while a single "case" sample runs the outlier analysis? An option to override this default behaviour could be offered for cases such as C9orf72.

Your point is well taken and we will continue to work on making EHdn easier to use. We wanted to provide the option of applying the outlier analysis to rare disease cohorts where only a small fraction of patients would be expected to have an expansion. We have extended documentation to make it easier for the users to decide between outlier and case-control analysis:

https://github.com/Illumina/ExpansionHunterDenovo/blob/master/documentation/01_Be

fore_you_begin.md#choosing-between-casecontrol-and-outlier-analysis

> 5) The final output consists solely of two summary tsv files. Given the potential for false positives, users ideally should be able to investigate all the reads that went into making this call. Many other variant detection tools (particularly SV tools) create new BAM files for each event allowing users to examine the evidence themselves however I appreciate in this instance many reads will be unaligned. While a constructed BAM file would be preferable, at a minimum a list of supporting reads used for each RE call should be provided.

This is an excellent idea. The “profile” command now outputs names, motifs, and mapping positions of all informative read pairs. This change is documented here: https://github.com/Illumina/ExpansionHunterDenovo/blob/master/documentation/05_Co

mputing_profiles.md#supplementary-files-generated-by-the-profile-command

We also considered the possibility of outputting a BAM file with all informative read pairs. This turned out to have a very significant impact on the memory usage (because of the need to hold information about each read in memory until read’s mate is seen). Instead, we implemented a script for extracting reads around any repeat region:

https://github.com/Illumina/ExpansionHunterDenovo/blob/master/documentation/05_Co

mputing_profiles.md#generating-a-bamlet-for-a-given-repeat-region

The script extends a given region (+/-2Kb), extracts reads from the extended region, and then extracts mates of the collected reads located anomalously far away (>1Kb). This script offers a way to investigate the reads that went into making a particular call.

> 6) Is there any handling of base calling errors within repeats? Given that the error rate will likely be higher than normal in these low complexity regions and the repeats potentially very long, is this handled and if so how?

Yes, we use what we call a “weighted purity score” that penalizes mismatches at lowquality bases less than mismatches at high-quality bases. We found that measures like the weighted purity score are important for addressing the elevated base-call error rate in highly repetitive regions of the genome.

> 7) Several other RE software packages are mentioned throughout the text for comparison noting limitations, differences, etc. It would help clarify where EHdn fits in with existing software (and previous EH iterations) by providing a comprehensive table highlighted the novelty of EHdn.

Thank you for suggesting this. Per the reviewer’s suggestion, we have now included this information in the Supplementary Table S8 which is an extended version of Table 2 from Bahlo et al., Recent advances in the detection of repeat expansions with shortread next-generation sequencing, <https://www.ncbi.nlm.nih.gov/pubmed/29946432>

> 8) EHdn is designed to detect expansions larger than the read size however presumably users are interested in detecting all expansions independent of size. Are there plans to handle this use case or else other tools to recommend such as previous EH versions?

Yes, we do plan to enable EHdn to detect repeat expansions shorter than the read length. This will require a significant amount of development to both code (to keep the runtime reasonable) and the statistical methods for comparing STR profiles (to deal with much larger set of regions to compare). Meanwhile, the existing targeted STR genotypers could be used to search for short repeat expansions among the already annotated STRs.

> 9) Most NGS sequencing technologies perform sub-optimally in high/low GC content regions which are often present in expansions. In your analysis what REs were validated using Sanger sequencing? Were any of the control population REs validated?

Yes, repeat expansions were experimentally confirmed in all 91 samples from the Coriell Institute. To further assess the accuracy of EHdn, we generated an STR profile for the Venter/HuRef genome and then checked if the signatures of the identified repeats are also present in PacBio long-read data. This analysis validated 77% of repeats supported by two in-repeat reads; the confirmation rate increases to 90% for repeats with more read support (see the Supplementary Figure S4 and the Supplementary Section "Validation with an orthogonal sequencing technology").

> 10) What happens if the same repeat expansion pattern is present at two distinct genomic locations? Would this be reported as one or two events? How will paired IRRs be handled in this case? Will these be counted twice?

In this scenario both loci will be identified because there will be IRRs that “anchor” at each location. The paired IRRs will be identified but not assigned to any specific location. In general, EHdn reports the overall count of paired IRRs allowing to detect very long expansions but not necessarily to place them in any specific region of the genome.

> Minor:

>

> 1) The output files columns are not described in the documentation. While mostly intuitive, these should be described. Also, is it possible to output a more widely acceptable format like vcf as well?

Thank you for noticing a gap in the documentation. We have now documented the

output files here:

https://github.com/Illumina/ExpansionHunterDenovo/blob/master/documentation/03_Ca

se_control_quickstart.md

https://github.com/Illumina/ExpansionHunterDenovo/blob/master/documentation/04_O

utlier_quickstart.md

We also extended the "profile" command to output a table with repeat coordinates,

motifs, and an approximate repeat size estimates:

https://github.com/Illumina/ExpansionHunterDenovo/blob/master/documentation/05_Co

mputing_profiles.md#supplementary-files-generated-by-the-profile-command

We are planning to add support for the VCF files later this year by introducing a tool that would piece together the structure of each repeat region identified by EHdn from short reads (making it possible to produce VCF records with single-nucleotide accuracy).

> 2) Fig 3: In addition to the four known expansions, there appear to be other significant regions. Were these investigated and if so what was found?

We have not performed detailed studies of these regions yet. We hope that the release of EHdn will encourage further studies aimed at characterizing such regions in very large populations. Undoubtedly, we still have much to learn about the biology of large repeats.

> 3) For users working with an increasingly large number of WGS samples it would be useful to have the option to run EHdn on targeted regions. Is it possible to add this feature?

Thank you for suggesting this. We implemented a way to restrict the analysis to a set of regions in a BED file during case-control and outlier analysis through the --targetregions command line parameter. See

https://github.com/Illumina/ExpansionHunterDenovo/blob/master/documentation/03_Ca

se_control_quickstart.md

https://github.com/Illumina/ExpansionHunterDenovo/blob/master/documentation/04_O

utlier_quickstart.md

We also considered adding a way to restrict the “profile” command to a specified set of regions. However this turned out to be more nuanced than we expected: In-repeat reads often get misaligned to other regions of the genome making their recovery less efficient than streaming the entire BAM file through EHdn.

> 4) Are controls samples required to run the EHdn?

The “profile” command can be used to obtain locations of STR profiles for a single sample. The supplementary files that the “profile” command now outputs, make the information about long repeats more accessible without running the outlier or case/control comparison:

https://github.com/Illumina/ExpansionHunterDenovo/blob/master/documentation/05_Co

mputing_profiles.md#supplementary-files-generated-by-the-profile-command

We also now provide a link in the documentation to the Polaris Diversity Cohort, a set of freely-available control samples:

https://github.com/Illumina/ExpansionHunterDenovo/blob/master/documentation/01_Be

fore_you_begin.md

> 5) Typo P16 L4 "of motif not present the reference genome"

Thank you. We have fixed this.

**Second round**

**Reviewer 1**

Authors have addressed my comments.

**Reviewer 2**

All concerns are adequately addressed. I recommend for publication.
